# Goldenhar Syndrome and Surgical Reconstruction: A Case Report of Bilateral Complete Eyelid Colobomas in a 2-Day-Old Patient

**DOI:** 10.1155/crop/6640462

**Published:** 2025-03-21

**Authors:** Rawan S. Utt, Suad M. Udwan, Waed Amro, Safaa Abatli, Saja Saadeh Issa, Bashar M. Y. Jaber

**Affiliations:** ^1^Al-Quds University Research Assistant, Opthalmology Resident at Jordan Hospital University, Amman, Jordan; ^2^Faculty of Medicine, Al-Quds University, Hebron, State of Palestine; ^3^Faculty of Medicine, An-Najah National University, Nablus, State of Palestine; ^4^Faculty of Medicine, Al-Quds University, Qalqilya, State of Palestine; ^5^Oculoplastic Surgeon at St John Eye Hospital, Jerusalem, State of Palestine

## Abstract

Goldenhar syndrome (GS), also known as Franceschetti–GS, encompasses a spectrum of congenital anomalies affecting the eyes, ears, face, and vertebrae. This case report highlights a 2-day-old female patient diagnosed with GS presenting a rare manifestation of bilateral complete eyelid colobomas. The patient, with associated renal and cardiac problems, underwent surgical interventions, including bilateral lower lid frost suture tarsorrhaphy and subsequent upper lid reconstructions. Despite challenges and complications, the patient showed varying degrees of improvement in corneal conditions postsurgery. The discussion provides insights into the clinical features, diagnosis, and multidisciplinary management of GS. The presented case emphasizes the importance of tailored surgical approaches in addressing the complex ocular manifestations of GS, aiming for functional and aesthetic outcomes. Ongoing follow-up and further reconstruction surgeries are planned to optimize visual outcomes and address residual complications.

## 1. Introduction

Goldenhar syndrome (GS), also known as Franceschetti–GS, facio-auriculo-vertebral spectrum (FAV), first and second branchial arch syndrome, or oculoauriculovertebral (OAV) syndrome, is a congenital disorder encompassing a spectrum of anomalies affecting the eyes, ears, face, and vertebrae. Ophthalmic manifestations of GS include unilateral or bilateral eyelid colobomas, choroidal or iridial colobomas, epibulbar dermoid and/or lipodermoid, anophthalmia, microphthalmia, and iris atrophy [1].

Eyelid coloboma represents a congenital, full-thickness defect in the eyelid margin, falling within the spectrum of craniofacial clefting. This defect ranges from a minor notch to a complete absence of the eyelid margin. While commonly situated between the medial and middle thirds of the upper eyelid, it can manifest in various locations. This anomaly may appear as a simple isolated feature or be part of a broader syndrome, as observed in other reported cases of GS [2]. Eyelid colobomas are one of the few ophthalmic conditions that require intervention at an early age. Management varies based on size, presence of corneopalpebral adhesions (CPAs), and completeness of the coloboma [3].

Herein, we present a unique case of GS, a 2-day-old female with bilateral complete eyelid colobomas, with no significant family history, managed surgically through comprehensive reconstruction to address the absent eyelid, highlighting the importance of timely intervention in such cases.

## 2. Case Presentation

**Figure 1:**
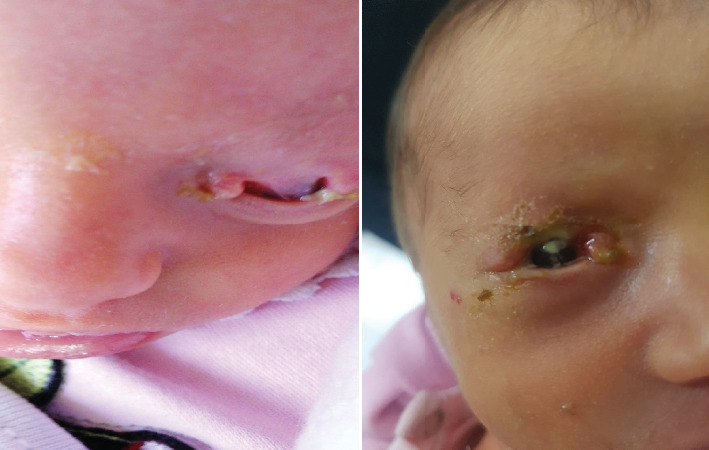
The patient at the age of 2 days with complete bilateral upper eyelid colobomas.

A 2-day-old female of first-degree cousin parents was referred by a pediatrician to our specialized ophthalmology hospital due to complete bilateral upper eyelid colobomas as shown in Figure 1, at the age of 2 days. The patient was found to have an atrial septal defect, which closed spontaneously later. Transfrontal ultrasound and abdominal ultrasound were done at 3 days of age and showed attenuated cerebrospinal fluid spaces and a marked dilatation of the left pelvicalyceal system, respectively. Upon ocular examination, both eyes revealed a clear corneal surface without ulcers or defects. Considering the combination of ocular, cardiac, and renal abnormalities, a diagnosis of GS was established; the patient's mother did not get a detailed US during pregnancy, so the diagnosis was not identified until after birth.

Two days later, bilateral corneal infiltrations developed, prompting examination by our pediatric ophthalmologist. Despite fortified antibiotic treatment, no improvement was observed. The oculoplastic consultant's report indicated extensive bilateral upper lid colobomas (more than 50%), accompanied by lid defects featuring CPAs, corneal exposure, and ulcers.

**Figure 2:**
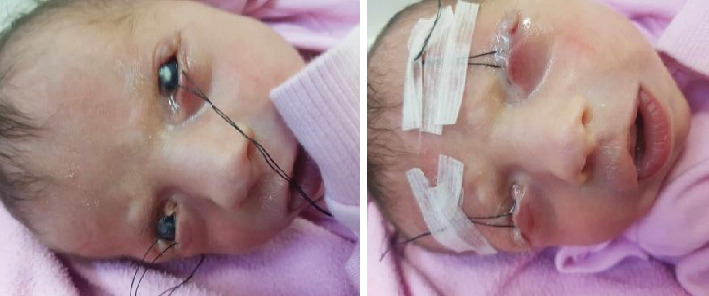
The patient at the age of 15 days after bilateral lower lid frost suture tarsorrhaphy with tapping to the forehead was done.

Referral to a corneal specialist for frost suture tarsorrhaphy was recommended but could not be executed due to the patient's unstable cardiac anomaly. At 15 days of age, the patient was transferred to another hospital, where bilateral lower lid frost suture tarsorrhaphy with tapping to the forehead was performed under general anesthesia as shown in Figure 2. Postsurgical care included instructions for tape release, eye drops, and visual stimulation. The third-day checkup revealed improvement in the right eye, while the left eye maintained its preoperative condition. Topical antibiotics, lubricants, and ointments were continued.

**Figure 3:**
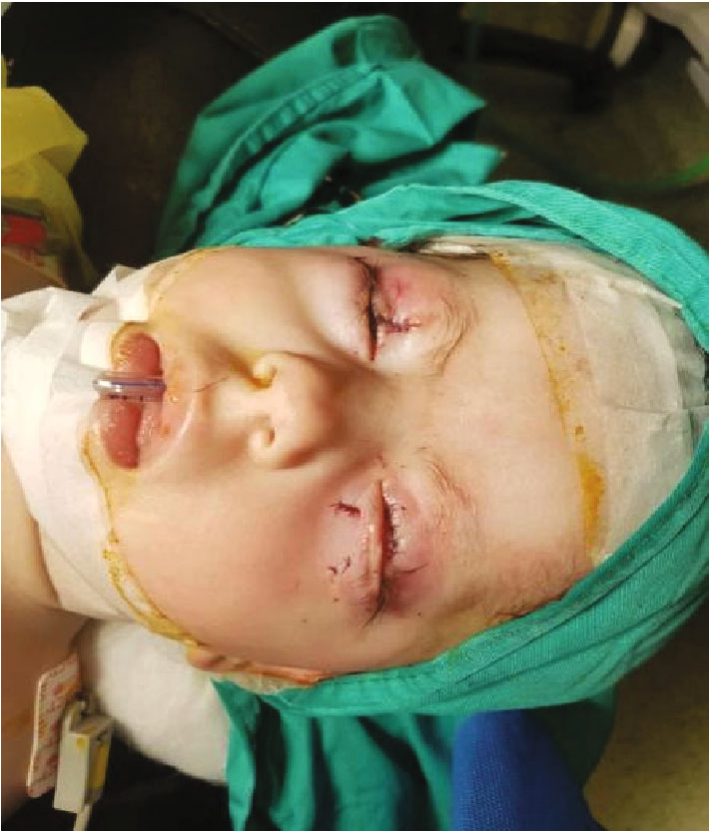
The patient at the age of 8 months after bilateral upper lid reconstruction (right Tenzel flap, left Cutler–Beard flap, and bilateral release of symblepharon and left mucous membrane graft).

By 40 days, the infection had resolved, leaving bilateral corneal scars. At 8 months, the patient underwent bilateral upper lid reconstruction involving a right Tenzel flap, a left Cutler–Beard flap, and bilateral release of symblepharon with a left mucous membrane graft as shown in Figure 3. A subsequent reconstruction surgery, comprising right upper lid notch repair and Stage 2 Cutler–Beard under general anesthesia, occurred a month later.

At 14 months, follow-up revealed successful correction on the right side with good upper lid contour and mild corneal haze. However, the left upper lid exhibited a central upper lid notch with a bulky central part and a dense corneal scar greater than the right upper lid. Regular follow-up continued.

**Figure 4:**
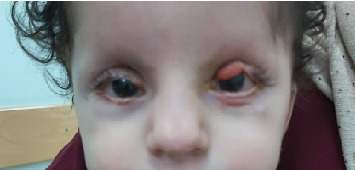
The patient at the age of 2 years after LT upper lid fourth reconstruction surgery (debulking of central upper lid).

**Figure 5:**
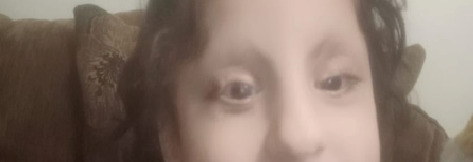
The patient at the age of 4 years has marked improvement cosmetically and normal visual acuity.

At 2 years, a fourth reconstruction surgery for the left upper lid was performed under general anesthesia, focusing on debulking the central upper lid as shown in Figure 4. The last checkup at 3 years and 4 months demonstrated a well-leveled and contoured right side with mild corneal haze, as shown in Figure 5. On the left side, the lower lid level showed upper ectropion and a corneal scar, leading to the decision for further reconstruction surgery after 6 months.

## 3. Discussion

The incidence of GS is reported to be 1:35,000–1:56,000, with a male-to-female ratio of 3:2 [4]. It is present in 1:1000 patients with congenital deafness [5]. Abnormalities are typically unilateral in about 85% of cases and usually affect the right side of the body. The syndrome is sporadic in most cases, but 1%–2% of cases have a family history, suggesting an autosomal dominant or autosomal recessive inheritance. Sporadic cases often involve 5p deletions, 14q23.1 duplications, and abnormalities of chromosomes 18 and 22 [6–8].

Research indicates that various genetic and environmental factors contribute to GS's development. Maternal use of certain drugs during pregnancy, such as thalidomide, retinoic acid, tamoxifen, and cocaine, has been linked to syndrome development. Additionally, maternal diabetes mellitus, TORCH, and influenza infections during pregnancy are reported etiological factors [6]. However, the presented case lacked these risk factors.

The clinical features of GS exhibit wide variability, necessitating long-term follow-up for accurate prognosis. Facial and auricular manifestations are prominent, including facial asymmetry, hemifacial microsomia (80%–99% of cases), preauricular skin tags, maxillary hypoplasia, cleft lip and palate, malar flattening, cheilopalatoschisis, and posteriorly rotated and low-set ears, among others. Bone manifestations involve hemivertebrae, fused vertebrae, scoliosis, rib, and extremity anomalies. Congenital heart defects are reported in 5%–58% of patients, comprising ventricular septal defect, tetralogy of Fallot, and transposition of great vessels [6]. Neurological anomalies such as microcephaly, encephalocele, hydrocephaly, and Arnold–Chiari malformation have been documented, with normal IQ observed in patients [4, 9, 10]. Gastrointestinal and urogenital tract anomalies, such as ectopic kidney and imperforate anus, are also reported. Ophthalmologically, the syndrome may present with upper eyelid, iris, and chorioretinal coloboma; subconjunctival dermoid; epibulbar choristoma; microphthalmia; anophthalmia; strabismus; and cataract [4, 9].

Diagnosis relies on clinical evaluation and radiologic findings. Prenatal detection through ultrasonography is feasible, revealing varying degrees of prevalent unilateral underdevelopment of craniofacial structures and spinal anomalies. The syndrome was diagnosed in a fetus at 15 weeks gestation, observing a maxillary cleft in association with unilateral microphthalmia [11–13].

GS lacks a standardized surgical management guideline due to its diverse manifestations and systemic associations. Treatment, ranging from cosmetic to complex reconstructive surgeries, requires an individualized approach adapted to age and disease severity. Neonatal focus involves evaluating vital functions, with cheiloplasty beginning before 3 months old. For moderately affected patients aged 2–4 years, no treatment may be necessary, but bone distraction devices and rib grafts are options for mandibular aplasia. Auricular reconstruction typically occurs after 8 years, focusing on the optimal chondral skeleton. Facial asymmetry is addressed between 8 and 10 years with lipoinjection of abdominal fat. In terms of the overall appearance of the patient, this may be the most important stage in the entire treatment programme [11, 14].

Eyelid coloboma necessitates immediate clinical–surgical attention, with corneal protection through therapeutic contact lenses or eye lubricating drops. Palpebral reconstruction is paramount for both functional restoration and aesthetic considerations. The timing of surgery depends on many factors, such as the coloboma's size, location, risk of corneal exposure, and the child's overall health. In cases of small defects without corneal exposure, surgery may be delayed. The surgical strategy ensures no alteration impacts the eyelid's static, dynamic, or aesthetic properties. Suturing is suitable for small defects, whereas more intricate techniques like the Cutler–Beard technique, Mustard rotational flap, tarsomarginal grafts, and modified Hughes procedure are employed for larger colobomas [10].

Managing complete colobomas poses a significant challenge as the surgeon's responsibility is twofold—creating a fornix and reconstructing the eyelid. The upper eyelid can be fully restored using a bipedicled cutaneous flap from the lower eyelid, ensuring the preservation of the orbicularis oculi muscle to prevent ectropion [15].

For Grade I pediatric limbal dermoids, the current medical approach is conservative. This involves a combination of excision, lamellar keratoplasty, amniotic membrane, and limbal stem cell transplantation in more advanced stages (II and III). Simple excision and conjunctivoplasty are primarily undertaken for lipodermoids with a focus on cosmetic improvement [10].

Severe kyphoscoliosis, carrying a high risk of neurologic impairment, necessitates osteotomies. Patients requiring posterior vertebral column resection surgery exhibit more severe and intricate defects [16].

The prognosis in GS cases is influenced by systemic involvement. However, cases without systemic associations generally have a favorable outlook. The categorization system recently introduced by Tasse et al. proves user-friendly and practically applicable, aiding in patient prognosis and classification [17, 18].

## 4. Conclusion

This case report focuses on the surgical management of a complex case of GS, particularly focusing on the ocular manifestations of bilateral complete eyelid colobomas. Despite challenges, the presented interventions demonstrated a variable degree of success in improving corneal conditions. The discussion provides an understanding of GS, emphasizing the importance of ongoing follow-up and the necessity for individualized treatment plans tailored to age and disease severity.

## Data Availability

Data is available upon request.
